# Prevalence and Prognosis of Atenolol-Responsive Systolic Anterior Motion of the Septal Mitral Valve Leaflet in Young Cats with Severe Dynamic Left Ventricular Outflow Tract Obstruction

**DOI:** 10.3390/ani12243509

**Published:** 2022-12-12

**Authors:** Michelle Kortas, Viktor Szatmári

**Affiliations:** Department of Clinical Sciences, Faculty of Veterinary Medicine, Utrecht University, Yalelaan 108, 3584 CM Utrecht, The Netherlands

**Keywords:** beta receptor blocker, β-blocker, congenital, echocardiography, feline, HOCM, hypertrophic cardiomyopathy, mitral regurgitation, mitral valve dysplasia, murmur, owner compliance, SAM

## Abstract

**Simple Summary:**

The most common heart disease in adult cats is hypertrophic cardiomyopathy (HCM), which is a slowly progressive, uncurable disease. Most cats with HCM live several years without obvious problems. However, several cats eventually develop life-threatening clinical signs or die suddenly. Hypertrophic cardiomyopathy is difficult to diagnose in clinically healthy cats because of the absence of any abnormalities on physical examination. However, several cats with HCM develop a heart murmur, which veterinarians can pick up at routine health checks. The reason for the murmur in many cats is an abnormal movement of a valve, which causes a leak and an obstruction. These findings can be visualized with cardiac ultrasonography. Noteworthy, the same ultrasonographic findings can be encountered in cats that do not have HCM but have an abnormality developed valve instead. An important difference between these two conditions is that some of the latter cases might be cured with life-long administration of a beta-blocker. The present study showed that the number of cats that benefited from atenolol therapy was disappointingly low. The reason why we examined only young cats with a heart murmur was that HCM occurs more frequently in elderly cats, whereas the potentially curable congenital disorder is present from birth.

**Abstract:**

Background: Severe dynamic left ventricular outflow tract obstruction (DLVOTO) secondary to the systolic anterior motion of the septal mitral valve leaflet (SAM) can result either from congenital mitral valve disorders or left ventricular concentric hypertrophy of any cause, in cats commonly hypertrophic cardiomyopathy (HCM). Though HCM cannot be reversed, the question remains whether atenolol can cure cats with severe DLVOTO resulting from a presumed mitral valve dysplasia. Methods: In this retrospective case series, client-owned asymptomatic cats younger than 1.5 years with echocardiographic evidence of SAM and severe DLVOTO were included. Oral atenolol therapy and recheck echocardiography after 2–3 months were recommended. The owners and referring veterinarians were called for long-term follow-up information. Results: Of the 28 included cats, 23 were treated with atenolol. Recheck echocardiography performed in 17 cats showed a resolution of SAM in 47%. In the long term, SAM remained absent in only 9% of the treated cats. Cardiac-related death occurred in 26% of the atenolol-treated cats. Conclusions: The long term benefit of twice-daily atenolol therapy was documented in 9% of cats. Whether the cats where atenolol failed to resolve DLVOTO on long-term had HCM, or a therapy-resistant congenital primary mitral valve disorder remains unclear.

## 1. Introduction

The systolic anterior motion of the mitral valve (SAM) is defined as the systolic displacement of the septal mitral valve leaflet into the left ventricular outflow tract (LVOT), causing mitral valve regurgitation and dynamic left ventricular outflow tract obstruction (DLVOTO) [1]. Systolic anterior motion can be caused by left ventricular concentric hypertrophy (cLVH) of any cause or by a primary congenital disorder of the mitral valve apparatus [2,3,4,5,6,7]. 

Concentric left ventricular hypertrophy can be either primary or secondary. The most common cause of primary cLVH in cats is hypertrophic cardiomyopathy (HCM) [3]. Secondary cLVH results from chronic pressure overload, e.g., due to systemic arterial hypertension or aortic stenosis. Aortic stenosis can be fixed or dynamic. The systolic anterior motion of the mitral valve can lead to a secondary cLVH through a severe DLVOTO, which is often considered a dynamic form of subaortic stenosis. Left ventricular concentric hypertrophy and SAM can coexist, where either of those can be the primary disorder. The simultaneous presence of HCM and SAM is known as the obstructive form of hypertrophic cardiomyopathy (HOCM) [1,4].

Oral atenolol therapy might resolve SAM by its negative inotropic effect in some cats and in young dogs with severe DLVOTO [5,6,7]. If the DLVOTO is resolved or its severity is decreased, the cLVH will resolve or decrease too (reverse remodeling), in case it was caused by chronic pressure overload. When SAM is left untreated, the resulting DLVOTO can cause pressure overload and, in turn, cLVH. If SAM is not resolved, the same consequences and prognosis apply as those for HOCM. Severe cLVH in cats can lead to (1) dyspnea as a result of cardiogenic pulmonary edema or pleural effusion secondary to congestive left-sided heart failure, (2) peracute limb paresis or paralysis as a result of ischemic myopathy secondary to arterial thromboembolism, (3) syncope as a result of arrhythmias or (4) sudden cardiac death [3].

If both SAM and cLVH are visible on echocardiography in a young cat, and the DLVOTO is severe, the echocardiographic differentiation of HOCM from a suspected congenital mitral valve disorder can be challenging, if not impossible. However, this differentiation might influence the therapy recommendation and the long-term prognosis. Atenolol cannot alter the natural history of HOCM [3,8], but it might resolve SAM, DLVOTO, and secondary cLVH in certain cats with suspected mitral valve dysplasia, as this has been documented in young dogs [6]. If severe DLVOTO is present without cLVH in a young cat, a primary congenital mitral valve disorder seems to be more likely than a HOCM.

According to the authors' knowledge, there is no literature available on the short- and long-term effects of chronic oral atenolol therapy in young cats with severe DLVOTO, where SAM is unlikely to be the result of a primary myocardial disorder, such as HCM. The aims of this retrospective study were (1) to establish how often atenolol could resolve SAM and DLVOTO on short-term in young cats with severe DLVOTO and (2) to provide long-term follow-up data on these cats. Our hypothesis was that SAM and severe DLVOTO caused by a congenital primary mitral valve disorder, presumed mitral valve dysplasia, can be resolved with chronic twice-daily administered oral atenolol therapy, and these cats would have a favorable long-term prognosis without cardiac-related morbidity and mortality.

## 2. Materials and Methods

### 2.1. Animals

For this retrospective case series, the electronic database of the authors’ institution was searched for client-owned asymptomatic cats younger than 1.5 years of age in a period of 15 years, between 2006 and 2021. All cats had to be referred to the cardiology service for evaluation of a murmur. Physical examination and echocardiography had to be performed by a board-certified (ECVIM-CA) cardiologist. Echocardiographic evidence of SAM and a DLVOTO with a minimum Doppler-derived peak pressure gradient of 75 mmHg (i.e., minimum peak blood flow velocity of 4.35 m/s) in an unsedated cat without any medical therapy had to be present to include a cat in the study. Cats were not included if the severity of the DLVOTO was less than 75 mmHg or if other congenital heart diseases than the SAM, such as fixed aortic stenosis, were found. Furthermore, cats had to be free of any clinical signs and not receive any medication at and prior to the initial examination. The reason for choosing 1.5 years of age as a cut-off was to reduce the chance for HOCM, which is increasingly common with older age [3]. Further reducing the cut-off age would have resulted in fewer cases. The reasons to include asymptomatic cats only were to have a more uniform sample, to exclude the effect of possible drug interactions and concerns about administering atenolol to cats with advanced heart disease if they were symptomatic due to congestive heart failure [3]. Cats, whose owners decided not to start with the recommended atenolol therapy, were not included in the study. Cats were excluded from the study if no follow-up information was available. 

### 2.2. Examinations

All cats underwent a physical examination and an echocardiogram performed by a board-certified (ECVIM-CA) veterinary cardiologist at the authors’ institution. The echocardiogram was carried out on unsedated animals manually restrained in right and left lateral recumbency. The echocardiographic examination consisted of 2-dimensional, M-mode, color Doppler and spectral (pulsed- and continuous wave) Doppler modes according to reported standards [9]. The severity of DLVOTO was determined by continuous wave Doppler mode using the modified Bernoulli equation after measurement of the peak blood flow velocity in the LVOT from the standard left parasternal 5-chamber echocardiographic view [9]. Severe DLVOTO was defined as a Doppler-derived pressure gradient of higher than 75 mmHg (i.e., minimum peak blood flow velocity of 4.35 m/s). The left ventricular wall thickness of the interventricular septum and the left ventricular free wall at end-diastole were measured using M-mode echocardiography in a right parasternal short-axis view at the level of the chordae tendineae [3]. A left ventricular free wall or septal thickness of <5.0 mm was considered normal and cLVH was considered present if it measured ≥6.0 mm. The hypertrophy was considered mild, if it measured 6.0–6.5 mm, moderate, if it measured 6.5–7.0 mm and severe, if it measured > 7.0 mm [10]. Left ventricular free wall or interventricular septal thickness between 5.0–6.0 mm was interpreted in the context of body weight [3,11,12]. Cats weighing 5–8 kg had a cut-off value of 5.5 mm as normal, and cats weighing <5 kg had a cut-off value of 5.0 mm [11,12].

### 2.3. Follow-Up

Atenolol was prescribed as an oral tablet with a dosage of 6.25 mg/cat q12h to cats above 4 kg bodyweight, or as oral liquid with an aimed dose of 1.5 mg/kg q12h to cats less than 4 kg bodyweight, after weekly up-titration with 0.5 mg/kg increments, PO. The dose of atenolol was increased from 6.25 mg to 12.5 mg/cat q12h in cats where recheck echocardiography 2–3 months later showed insufficient reduction in the severity of DLVOTO with the persistence of SAM. The severity of DLVOTO and the presence of SAM were the only variables that influenced the attending cardiologist’s decision on whether to increase the atenolol dose. Heart rate, murmur presence and intensity, or the severity of cLVH on echocardiography did not play a role in the decision making. If owners stopped atenolol therapy for whatever reason, their cats were not excluded from the study.

Baseline characteristics of the cats were collected at presentation to the authors’ institution, such as the age, the intensity of the heart murmur, and the presence and severity of cLVH. Atenolol therapy was recommended only if the severity of DLVOTO was above 75 mmHg. The final decision whether to start with the atenolol in these cats was at the discretion of the owner, after understanding that this treatment might mean a life-long (i.e., often years) administration of twice daily oral medication with an uncertain clinical benefit.

Owners were called in November 2021 to collect the following data: (1) whether the cat was still alive, if not when and how the cat died, (2) if the cat was alive whether atenolol was still administered, if atenolol was stopped the reason was asked, (3) whether the cat was clinically healthy, if not what kind of clinical signs were present and for how long. In addition, the referring veterinarians of all cats were called to ask about the presence of a cardiac murmur at the last consultation.

Atenolol responsiveness was defined as resolution of SAM and DLVOTO on recheck echocardiography 2–3 months after the start of the therapy, while the cat was receiving oral atenolol therapy. 

Cardiac-related death was defined as sudden death or euthanasia prompted by clinical signs consistent with congestive left-sided heart failure or arterial thromboembolism [3].

### 2.4. Statistical Analysis

For descriptive statistics, counts, proportions or medians with ranges were reported. Normal distribution of data was calculated by the Shapiro-Wilk test. Survival time was calculated from the start of atenolol therapy to the date of death or last follow-up. Cats that were still alive at the last follow-up were censored. The prevalence is expressed as percentage of cats where SAM and DLVOTO resolved. Statistical analysis was performed using a commercially available software (SPSS Statistics version 27, IBM, New York, NY, USA).

## 3. Results

### 3.1. Animals

An electronic search in the database resulted in 60 cats with SAM that were younger than 1.5 years of age at first presentation. Of these 60 cats, 6 cats were not included because they showed clinical signs at presentation, and 5 cats were not included because echocardiography revealed more than one congenital cardiac anomaly. An additional 16 cats were not included because the pressure gradient of their DLVOTO was less than 75 mmHg. The owners of the remaining 33 cats were called, of which 3 owners could not be reached via telephone or email and 2 owners did not want to share information about their cats. Of the remaining 28 cats with a minimum Doppler-derived pressure gradient of 75 mmHg at the initial echocardiography, 23 cats were treated with atenolol and 5 owners declined to start with the recommended therapy. 

The median age of the 23 cats at their first echocardiography at the authors’ institution was 10 months (range 3–17 months). Further characteristics of the cats are presented in Table 1.

### 3.2. Treatment Duration, Dosage Changes and Reasons to Stop Therapy

Of the 23 cats, 20 cats were started at a dosage of 6.25 mg atenolol/cat q12h. The dosages of 16 of these cats did not change over time, according to their owners. The duration of the therapy from the start of treatment to the telephone interview was a median of 4.8 years (range 2 months–10.6 years).

In 2 cats, the dose was increased from 6.25 mg/cat q12h to 12.5 mg/cat q12h. In one of these cats, this change was recommended by the attending cardiologist at the recheck echocardiography after 3 months because 6.25 mg/cat q12h did not sufficiently reduce the severity of DLVOTO. In the other cat, the SAM was initially resolved after 10 days, but it was present at the third echocardiography performed almost 4 months after the first echocardiography. 

Atenolol dose was decreased in one cat from 6.25 mg/cat q12h to 6.25 mg/cat q24h in consultation with the referring veterinarian because ‘the cat was doing well’ and not because the owner found administering atenolol difficult. This happened 4 months after starting the treatment. 

There was one cat whose atenolol dosage was changed from 6.25 mg/cat q12h to 12.5 mg/cat q12h, then again back to 6.25 mg/cat q12h after 2 months. This was because, with the 6.25 mg/cat q12h dose, the severity of DLVOTO reduced but the SAM persisted. Since the doubled dose did not have a better effect on SAM, the atenolol dosage was changed back to 6.25 mg/cat q12h.

There were three cats that were started at a lower atenolol dose using the oral liquid, which was prepared by the pharmacy of the authors’ institution, because of the kittens’ small body weights at presentation (1.7, 2.8 and 2.9 kg). One of these three kittens received 1.5 mg/kg q12h, the second kitten received 0.5 mg/kg q12h for one week and thereafter 1.0 mg/kg q12h for 3 months, and the third kitten received 0.5 mg/kg q12h for one week, thereafter 1.0 mg/kg for one week, and 1.5 mg/kg q12h for one week, and thereafter they switched to tablets. Eventually, all these 3 cats received tablets (6.25 mg/cat q12h) when they reached their adult body weight (>4 kg).

Atenolol therapy was discontinued in 8 of the 23 cats (35%), with a median of 6 months (range 3 months–2.7 years) after starting the treatment. This was due to the following: (1) no or an insufficient effect of treatment (after a median of 3 months; range 3 months–1.3 years) on the severity of cLVH or DLVOTO, in consultation with a cardiologist or the referring veterinarian in 5 cats, or (2) the owners found it difficult or too stressful to administer the medication to the cat (after a median of 9 months; range 3 months−2.7 years) in the remaining 3 cats. None of the owners noticed any difference in their cats after stopping the oral atenolol treatment. Two owners indicated that it was sometimes difficult to administer the medication, but despite this challenge, they continued atenolol treatment. 

### 3.3. Severity of the Dynamic Left Ventricular Outflow Tract Obstruction and the Left Ventricular Concentric Hypertrophy on Echocardiography

Physical examination and echocardiography of the included cats were performed by four board-certified (ECVIM-CA) veterinary cardiologists.

At baseline echocardiography, the median severity of the DLVOTO was 117 mmHg (range 75–170 mmHg). 

At baseline echocardiography, there were four cats with normal left ventricular wall thickness, eight cats had a mild cLVH, four had a moderate cLVH and seven cats had a severe cLVH. 

### 3.4. Effect of Atenolol on the Presence of Murmur, Systolic Anterior Motion of the Mitral Valve and Severity of Dynamic Left Ventricular Outflow Tract Obstruction 

Of the 23 cats treated with atenolol, 6 cats did not undergo a recheck echocardiography. In two of these six cats, this was because the cat was not cooperative enough to perform the examination without sedation, in one cat, the owner canceled the appointment as she did not manage to get the cat into the carrier at home, the fourth cat’s owner did not come back because of financial restrictions, the fifth cat’s owner decided to have the recheck echocardiography performed elsewhere, and the sixth cat’s owner felt that the cat had too much stress administering the atenolol.

Therefore, 17 cats underwent a second echocardiography with a median of 3.5 months (range 10 days–17.8 months) later, at a median age of 11 months (range 5 months–1.8 years). In 8 of these 17 cats (47%), the SAM was resolved and the DLVOTO disappeared. In five of these eight cats, no audible murmur was present at the first recheck. Of these 17 cats, one cat underwent a third echocardiography 14 weeks after the second one. At this third echocardiography, the SAM was present again with a severe DLVOTO, while the cat was still receiving atenolol. 

In two of the five cats where the murmur disappeared at the first recheck, the murmur was still absent at the last known visit at their referring veterinarian. One of them was 1.5 years old and 6 months after the starting the atenolol therapy, and the other cat was 7 years old and 5.5 years after initiation of the atenolol therapy. This last cat was one of four cats that had a severe DLVOTO without cLVH at baseline echocardiography. In another treated cat with a severe DLVOTO and no cLVH at baseline echocardiography, SAM was also resolved, but information about the murmur at the last consultation by the referring veterinarian was unavailable because of missing documentation; and the owner did not know either whether the veterinarian heard a murmur. The other two treated cats did not return for a recheck. In these two cats, information about the murmur was also not documented by their referring veterinarians, but the owners reported during the telephone interview that their veterinarians mentioned the presence of a murmur at their last visits. However, the date of the last visit and whether this information was valid remain unknown. 

The median length of the follow-up period was 4.9 years (range: 2 months–10.6 years). Auscultation by the referring veterinarian at the last available follow-up revealed no murmur in 17% of the cats, a murmur in 74% of the cats and the presence of a murmur was unknown in 9% of the cats. 

The median time between the first and second echocardiography of the group of cats where the SAM resolved was 2.5 months (range: 10 days–4 months), whereas this time interval was 3 months (range: 2–16 months) for the group where the SAM did not resolve. In one cat the second echocardiography was performed already after 10 days at the owner’s request.

### 3.5. Murmur Intensity 

At enrollment, the median murmur intensity was 3 out of 6 (range 2–4, using Levine’s 6-scale system). 

The murmur intensity at the first visit in 7 out of 8 cats, whose SAM resolved later with atenolol, was 3 out of 6 and the remaining cat had a murmur intensity of 4 out of 6. The murmur intensity at the first visit before starting atenolol treatment of the 15 treated cats, whose SAM was not resolved with atenolol, was a median of 3 out of 6 (range 2–4): 3 cats had 4 out of 6, 10 cats had 3 out of 6 and 2 cats had 2 out of 6. No significant difference in murmur intensity at the initial visit was found (*p* = 0.834) when the group of cats whose SAM resolved with atenolol was compared to the group of cats whose SAM did not resolve. 

At the first recheck, in 5 of the 8 cats where the SAM was resolved, no audible murmur was present. In the remaining 3 cats, the median murmur intensity at the first recheck was 2 out of 6 (range 1–2). In these 3 cats, the murmur had a decreased intensity compared to the initial visit as follows: one cat had a murmur intensity of 1 out of 6, the second cat had 1–2 out of 6 and the third had a murmur intensity of 2 out of 6 only at a faster heart rate and at a slower heart rate, no murmur was audible 10 days after the start of atenolol therapy. However, after 4 months, the murmur was audible again, with a murmur intensity of 2 out of 6 in this latter cat. 

In 9 cats, the presence of SAM and the intensity of the heart murmur remained unchanged at the first recheck, with a median murmur intensity of 2 out of 6 (range 2–3). The murmur intensity decreased in 6 cats with one grade and in 2 cats the intensity remained unchanged. In one cat, the heart could not be evaluated with auscultation due to persistent purring.

Four of the 23 cats had no audible heart murmur at their last known visit to their referring veterinarians, whereas 7 cats still had a murmur with a documented intensity. Of the remaining 12 cats, information concerning the heart murmur was not documented. Of 10 of these 12 cats, the presence of murmur was documented at the last visit, but not the intensity. In 2 cats, both the presence and intensity were unknown. Of the 17 cats that had a documented heart murmur at their last visit with their referring veterinarian, 7 cats were not receiving atenolol when the referring veterinarian performed the cardiac auscultation.

### 3.6. Clinical Signs and Survival

In the study period, 6 of the 23 cats died (26%). All deaths were considered cardiac-related. Only one of these cats had a single episode of syncope during the treatment period before death. This cat had severe cLVH at initial echocardiography and still had severe cLVH at recheck echocardiography after 3 months. All the alive cats had not experienced any clinical signs at the time of the telephone interview.

One cat, whose SAM had resolved, was euthanized because of congestive left-sided heart failure. This cat died at 4.8 years of age, 3.3 years after the last check-up, where the resolution of SAM was documented. This cat had mild cLVH at initial echocardiography and still had mild cLVH at recheck echocardiography after 3 months. This owner stopped atenolol therapy after 2.7 years because it got more and more challenging to administer the pills. So, this cat received no atenolol for 6 months after stopping treatment before congestive heart failure occurred. 

Three cats died suddenly of presumed cardiac death, of which one had a documented resolved SAM. This last cat died at 5 years of age, while it was still receiving atenolol, 4.3 years after the last check-up where resolution of SAM was documented and after 4.5 years of atenolol therapy. This cat had no cLVH at initial echocardiography and still no cLVH at recheck echocardiography after 2 months. The second cat that died suddenly was 3.9 years old, 3.4 years after the last recheck whereby SAM was unresolved and after 3.4 years of continuous atenolol therapy until death. This cat had no cLVH at initial echocardiography, but no recheck echocardiography was performed because of its aggressive behavior. The third cat died at the age of 5.7 years, while it was still receiving atenolol, 3.8 years after the last consultation where SAM was unresolved and after 5 years of atenolol therapy. This cat had moderate cLVH at initial echocardiography and mild cLVH at recheck echocardiography after 2 months.

Two cats were euthanized because of clinical signs compatible with arterial thromboembolism. One of these cats died at the age of 4.8 years, while it was still receiving atenolol, 2.6 years after the last recheck, whereby SAM was still present, and after 4 years of atenolol therapy. This cat had severe cLVH at initial echocardiography and still severe cLVH at recheck echocardiography after 2 months. The other cat died at 3.4 years of age, while he was still receiving atenolol, 1.8 years after the last recheck whereby SAM was unresolved and after 2 years of atenolol therapy. This cat had severe cLVH at initial echocardiography and severe cLVH at recheck echocardiography after 3 months.

## 4. Discussion

The short-term prevalence of atenolol-responsive SAM was 47% in the present study, i.e., 8 of the 17 cats with severe DLVOTO had a resolution of SAM and DLVOTO at echocardiography after a median of 3 months of twice-daily oral atenolol therapy. In 30% of the cats (5 out of 17), the heart murmur also disappeared. In the 3 cats where SAM resolved but the murmur remained audible, an innocent or iatrogenic murmur was considered most likely, as echocardiography showed no blood flow velocities exceeding 2.0 m/s [13,14]. 

Atenolol therapy seemed effective in the long term in only 9% (2 out of 23) of the cats. In these cats, atenolol resolved the SAM and the severe DLVOTO at the recheck echocardiography, and no heart murmur was audible at the last documented visit at the referring veterinarian. In fact, this percentage might have been slightly higher, as only 74% of the atenolol-treated cats underwent a recheck echocardiography. One of these two cats had a normal left ventricular wall thickness, and the other had mildly thickened left ventricular walls at initial echocardiography, which made mitral valve dysplasia more likely than a primary myocardial disease such as HOCM. The cats with severe DLVOTO and normal left ventricular wall thickness at the initial echocardiogram were thought to have mitral valve dysplasia, similar to those young dogs that were described with atenolol-responsive DLVOTO and SAM [6]. Whether the cats that developed cardiac-related clinical signs had a therapy-resistant congenital mitral valve disorder causing the severe DLVOTO or they had a progressive myocardial disease, such as HOCM or myocarditis, remains unclear. It is also unknown whether the murmur, SAM and severe DLVOTO were present at the veterinary consultation only due to stress or whether these changes were present also at home without stress.

Six cats in the present study died, all from a cardiac-related cause. Though the owner of one cat stopped atenolol a year before its death, the rest of the cats were treated with atenolol until their deaths. All these cats had a murmur at the last known consult with their veterinarians. It remains unknown whether a new heart disease, like cardiomyopathy, developed or the original heart disease had worsened between the last echocardiography and the appearance of cardiac-related clinical signs.

There are several theories proposed for the mechanism of SAM in various mammalian species. The thickened interventricular septum can cause an altered blood flow in the LVOT pushing the septal leaflet of the mitral valve toward the interventricular septum in systole [1,6,15]. Elongated septal leaflets might also get caught by the flow and pushed towards the septum. Displaced papillary muscles of the mitral valve can pull the septal mitral valve leaflet into the LVOT as well [1,16]. Papillary muscle displacement can be either the result of geometrical changes of the left ventricle secondary to cLVH or it could be caused by a congenital malformation of the mitral valve apparatus, which can include anomalies not only of the valve leaflets but also of the chordae tendineae and the papillary muscles [1,17,18]. Treatment of severe DLVOTO with beta receptor blockers in cats is extrapolated from human cardiology [1,19]. In cats, experts believe that atenolol may reduce the severity of DLVOTO in asymptomatic cats with HOCM [20,21].

Whether SAM and DLVOTO would remain absent after discontinuation of atenolol treatment is unknown, as no recheck echocardiography was performed after atenolol was stopped. Therefore, it is unknown whether life-long administration of atenolol is necessary in atenolol-responsive cats, as SAM might resolve spontaneously during growth if SAM and DLVOTO were diagnosed in a young animal [6]. 

It is unclear why a heart murmur and DLVOTO returned in a number of cats in our study despite uninterrupted atenolol treatment. An increase in left ventricle contraction during stress may worsen SAM and DLVOTO [1,6,22,23]. Moreover, the upregulation of beta-1 adrenergic receptors with chronic atenolol therapy might have played a role. The progression of the underlying cardiac pathology, like in the case of HOCM, might also have contributed to the recurring of the murmur or worsening the murmur intensity and the severity of DLVOTO.

There is little to no scientific evidence about what atenolol dosage should be optimal for cats with SAM and severe DLVOTO. The current recommendation of 6.25–12.5 mg atenolol per cat PO every 12 h is based on practical considerations of dividing the available 25 mg human tablets into a quarter or a half [5,21,24]. There are several concerns about chronic atenolol therapy in cats. As atenolol affects not only the left ventricular but also the left atrial systolic function, it could facilitate thrombus formation; however, this is not thought to be clinically relevant [24,25,26]. More importantly, life-long, two-times-a-day oral medication of a cat can have a major impact on the quality of life of both the owner and the cat [21,27,28]. This should be considered when deciding whether to recommend a life-long therapy, as 22% of the owners in the present study found pilling their cats sometimes difficult or too stressful and 13%, therefore, even stopped the treatment.

In the present case series, an apparently wrong assumption was made by the cardiologists, namely that the DLVOTO would remain life-long absent if the recheck echocardiography 2–3 months after the initial examination and the start of atenolol therapy showed resolution of the SAM and the DLVOTO. Recommending at least yearly echocardiographic follow-up examinations to owners would probably help to adjust the atenolol dose and detect progressive cardiac disease in a preclinical stage. Though a recheck echocardiography can be stressful too, this is a once-a-year event as opposed to the possibly unjustified life-long twice-daily pilling.

The present study has a number of limitations. The small sample size and the low event rate (i.e., death) made it impossible to draw statistically sound conclusions on the long-term effects of atenolol in young cats with severe DLVOTO. Another limitation is the lack of regular (e.g., yearly) echocardiography recheck examinations until the first appearance of cardiac-related clinical signs (including sudden death). A further limitation is that the long-term follow-up examination findings were available only from the owners (regarding clinical signs) and from the referring veterinarians (regarding clinical signs and the presence of a murmur). Recording heart rate during echocardiography could have helped to assess the stress level of the cats, which is a known variable influencing the severity of DLVOTO. No blood tests were performed to assess potential changes in cardiac biomarker (i.e., serum troponin-I or plasma N-terminal pro-brain natriuretic peptide) levels as a response to the therapy. 

## 5. Conclusions

The present study showed that only 9% of cats seemed to have benefited from a long-term oral atenolol therapy when a severe DLVOTO caused by SAM due to a suspected congenital primary mitral valve disorder was diagnosed at a young age. Whether this low percentage reflects the low prevalence of congenital mitral valve disorder (i.e., primary SAM) or a lack of response to atenolol therapy, it remains unknown. Even though atenolol resolved SAM and the associated DLVOTO in some young cats with suspected mitral valve dysplasia, it did not seem to affect the long-term prognosis in most cats. Therefore, the question remains whether atenolol is a useful therapy in these young cats and also whether they had a congenital primary mitral valve disorder or an acquired progressive myocardial disease, like HOCM. 

## Figures and Tables

**Table 1:** Characteristics of 23 young cats with severe dynamic left ventricular outflow tract obstruction (DLVOTO) due to systolic anterior motion of the mitral valve.

**Breed (*n*)**	Domestic shorthair	19
	British shorthair	1
	Bengal	1
	Persian	1
	Sphynx	1
**Sex (*n*)**	Female	10
	Male	13
**DLVOTO severity at enrollment (median mmHg)**	117 (range 75–170)	
**Murmur intensity at enrollment (median scale 1–6)**	3 (range 2–4)	
**Follow-up (median)**	4.9 years (range 2 months–10.6 years)	
**Death (*n*)**	Cardiac death	6
	Non-cardiac death	0

*n* number of cats.

## Data Availability

The data that support the findings of this study are available from the corresponding author upon reasonable request.

## References

[1] Manabe S., Kasegawa H., Arai H., Takanashi S. (2018). Management of systolic anterior motion of the mitral valve: A mechanism-based approach. Gen. Thorac. Cardiovasc. Surg..

[2] Kuijpers N.W., Szatmari V. (2011). Mitral valve dysplasia in a cat causing reversible left ventricular hypertrophy and dynamic outflow tract obstruction. Tijdschr. Diergeneeskd..

[3] Luis Fuentes V., Abbott J., Chetboul V., Côté E., Fox P.R., Häggström J., Kittleson M.D., Schober K., Stern J.A. (2020). ACVIM consensus statement guidelines for the classification, diagnosis, and management of cardiomyopathies in cats. J. Vet. Intern. Med..

[4] Sherrid M.V. (2006). Pathophysiology and treatment of hypertrophic cardiomyopathy. Prog. Cardiovasc. Dis..

[5] Jackson B.L., Adin D.B., Lehmkuhl L.B. (2015). Effect of atenolol on heart rate, arrhythmias, blood pressure, and dynamic left ventricular outflow tract obstruction in cats with subclinical hypertrophic cardiomyopathy. J. Vet. Cardiol..

[6] Connolly D.J., Boswood A. (2003). Dynamic obstruction of the left ventricular outflow tract in four young dogs. J. Small Anim. Pract..

[7] Szatmári V. (2022). Congenital supravalvular aortic stenosis in a kitten. J. Vet. Cardiol..

[8] Coleman A.E., DeFrancesco T.C., Griffiths E.H., Lascelles B.D.X., Kleisch D.J., Atkins C.E., Keene B.W. (2020). Atenolol in cats with subclinical hypertrophic cardiomyopathy: A double-blind, placebo-controlled, randomized clinical trial of effect on quality of life, activity, and cardiac biomarkers. J. Vet. Cardiol..

[9] Thomas W.P., Gaber C.E., Jacobs G.J., Kaplan P.M., Lombard C.W., Moise N.S., Moses B.L. (1993). Recommendations for standards in transthoracic two-dimensional echocardiography in the dog and cat. J. Vet. Intern. Med..

[10] Wess G., Daisenberger P., Mahling M., Hirschberger J., Hartmann K. (2011). Utility of measuring plasma N-terminal pro-brain natriuretic peptide in detecting hypertrophic cardiomyopathy and differentiating grades of severity in cats. Vet. Clin. Pathol..

[11] Chetboul V., Petit A., Gouni V., Trehiou-Sechi E., Misbach C., Balouka D., Sampedrano C.C., Pouchelon J., Tissier R., Abitbol M. (2012). Prospective echocardiographic and tissue Doppler screening of a large Sphynx cat population: Reference ranges, heart disease prevalence and genetic aspects. J. Vet. Cardiol..

[12] Häggström J., Andersson Å.O., Falk T., Nilsfors L., OIsson U., Kresken J.G., Höglund K., Rishnew M., Tidholm A., Ljungvall I. (2016). Effect of body weight on echocardiographic measurements in 19,866 pure-bred cats with or without heart disease. J. Vet. Intern. Med..

[13] Ferasin L., Ferasin H., Kilkenny E. (2020). Heart murmurs in apparently healthy cats caused by iatrogenic dynamic right ventricular outflow tract obstruction. J. Vet. Intern. Med..

[14] Howell K.L., Ferasin L., Walls A., Smith N. (2022). Prevalence of iatrogenic heart murmurs in a population of apparently healthy cats. J. Small Anim. Pract..

[15] Sherrid M.V., Gunsburg D.Z., Moldenhauer S., Pearle G. (2000). Systolic anterior motion begins at low left ventricular outflow tract velocity in obstructive hypertrophic cardiomyopathy. J. Am. Coll. Cardiol..

[16] Seo J., Matos J.N., Payne J.R., Fuentes V.L., Connolly D.J. (2021). Anterior mitral valve leaflet length in cats with hypertrophic cardiomyopathy. J. Vet. Cardiol..

[17] Schrope D.P. (2015). Prevalence of congenital heart disease in 76,301 mixed-breed dogs and 57,025 mixed-breed cats. J. Vet. Cardiol..

[18] Jiang L., Levine R.A., King M.E., Weyman A.E. (1987). An integrated mechanism for systolic anterior motion of the mitral valve in hypertrophic cardiomyopathy based on echocardiographic observations. Am. Heart J..

[19] Ibrahim M., Rao C., Ashrafian H., Chaudhry U., Darzi A., Athanasiou T. (2012). Modern management of systolic anterior motion of the mitral valve. Eur. J. Cardiothorac. Surg..

[20] Gordon S.G., August J.R. (2006). Chapter 34-Cardiomyopathy—Therapeutic Decisions. Consultations in Feline Internal Medicine.

[21] Rishniw M., Pion P.D. (2011). Is treatment of feline hypertrophic cardiomyopathy based in science or faith? A survey of cardiologists and a literature search. J. Feline Med. Surg..

[22] Maron M.S., Olivotto I., Zenovich A.G., Link M.S., Pandian N.G., Kuvin J.T., Nistri S., Cecchi F., Udelson J.E., Maron B.J. (2021). Hypertrophic cardiomyopathy is predominantly a disease of left ventricular outflow tract obstruction. Circulation.

[23] Pellikka P.A., Oh J.K., Bailey K.R., Nichols B.A., Monahan K.H., Tajik A.J. (1992). Dynamic intraventricular obstruction during dobutamine stress echocardiography. A new observation. Circulation.

[24] Sugimoto K., Aoki T., Fujii Y. (2020). Effects of atenolol on left atrial and left ventricular function in healthy cats and in cats with hypertrophic cardiomyopathy. J. Vet. Med. Sci..

[25] Schober K.E., Zientek J., Li X., Fuentes V.L., Bonagura J.D. (2013). Effect of treatment with atenolol on 5-year survival in cats with preclinical (asymptomatic) hypertrophic cardiomyopathy. J. Vet. Cardiol..

[26] Riesen S.C., Schober K.E., Cervenec R.M., Bonagura J.D. (2011). Comparison of the effects of ivabradine and atenolol on heart rate and echocardiographic variables of left heart function in healthy cats. J. Vet. Intern. Med..

[27] Murphy L.A., Wang M.L., O’Malley B., Schrope D.P., Allen J.W., Chapel E.H., Russell N.J., Zimmerman S.A., Sloan C.Q., Nakamura R.K. (2022). A multi-center prospective evaluation of owner medication adherence for feline cardiovascular disease in the referral setting. J. Vet Cardiol..

[28] Taylor S., Caney S., Bessant C., Gunn-Moore D. (2022). Online survey of owners’ experiences of medicating their cats at home. J. Feline Med. Surg..

